# Adaptive multi-feature fusion architecture with optimized learning for high-fidelity brain tumor classification in MRI

**DOI:** 10.1038/s41598-026-38672-8

**Published:** 2026-03-09

**Authors:** Mohammed Safy, Mahmoud Khaled Abd-Ellah, Esraa Salah Bayoumi, Gerges M. Salama

**Affiliations:** 1 College of Computing and Information Technology, Arab Academy for Science, Technology and Maritime Transport (AASTMT), Smart Village, B 2401, Giza, Egypt; 2 Faculty of Artificial Intelligence, Egyptian Russian University, Cairo, 11829 Egypt; 3 Department of Electrical Engineering, Faculty of Engineering, Minia University, Minia, 61111 Egypt; 4 Electrical Engineering Department, Egyptian Academy for Engineering and Advanced Technology, Cairo, Egypt

**Keywords:** Image enhancement technique, Multi-class gliomas, Tumor classification, CGFF layers, Friedman test, Cancer, Computational biology and bioinformatics, Engineering, Mathematics and computing

## Abstract

Brain gliomas represent one of the most aggressive cancers worldwide and remain difficult to diagnose accurately at an early stage. Although computer-aided diagnostic (CAD) approaches have progressed notably in recent years, distinguishing between high-grade glioma (HG-G), low-grade glioma (LG-G), and healthy brain tissue on magnetic resonance images is still a major challenge. To address this issue, we propose a multi-stage framework designed to push the boundaries of current classification methods. The framework begins with a preprocessing phase that integrates Adaptive Gamma Correction (AGC) for improved contrast adjustment with a Denoising Convolutional Neural Network (DnCNN) for noise removal. Feature extraction is then carried out from three representative layers across three fine-tuned transfer learning CNNs (TRCNNs), where each model is optimized by a different algorithm. These deep representations are combined with handcrafted texture measures based on the Gray-Level Co-occurrence Matrix (GLCM), producing nine unique CNN–GLCM Fused Feature (CGFF) sets. The resulting hybrid descriptors are evaluated using several strong classifiers such as Random Forest (RF), Extreme Gradient Boosting (XGBoost), Light Gradient Boosting Machine (LightGBM), and Support Vector Machine (SVM), along with a stacked ensemble to reinforce stability and robustness. Performance significance was verified through the Friedman statistical test, with *p* < 0.05, confirming the reliability of the improvements. The framework achieved 99.05% accuracy, 98.99% recall, 99.52% specificity, 99.08% positive predictive value (PPV), and 99.54% negative predictive value (NPV), consistently surpassed state-of-the-art (SOTA) methods across all reported metrics.

## Introduction

The prompt and precise detection of the brain tumor grade directly influences the patient’s anticipated survival and plays a crucial role in treatment planning and evaluating tumor growth^1^. Significantly, brain tumors contribute to 85–90% of all central nervous system damage across all medical conditions^2^. Based on recent data provided by the National Brain Tumor Society (NBTS), one million Americans are living with a brain tumor. By the end of 2023, 94,390 cases of primary brain tumors were recorded, and 18,990 individuals died^3^. Gliomas are viewed as the most aggressive primary brain tumors within the central nervous system (CNS)^4^. The World Health Organization (WHO) classifies glioma tumors into two groups: low-grade (LG-G) and high-grade (HG-G)^5^. This classification relies on the microscopic appearance of tumor cells. Examination under the microscope reveals that cells in low-grade gliomas divide slowly. Although non-cancerous, low-grade gliomas can create issues by exerting pressure on normal brain structures. In contrast, high-grade gliomas exhibit more rapid and aggressive growth when compared to low-grade gliomas^6^.

Magnetic resonance imaging (MRI) is an advanced imaging technology known for its ability to offer detailed insights into brain tissue, making it a common choice for automated tumor detection^7^. MRI, which produces highly accurate images of brain tumor features, may provide patients with alternative therapy options. The high prevalence of brain tumors generates massive amounts of brain tumor MRI data, underscoring the urgent need for a reliable automated brain tumor diagnostic system^7,8^. Utilizing a Computer-Aided Diagnosis (CAD) system is crucial for swift and accurate brain tumor identification without human intervention^9^. The treatment approach for a brain tumor will differ based on the specific type of tumor. Automated detection of brain tumors through MRI scans can greatly enhance diagnosis and treatment planning. The process of automatic tumor diagnosis involves two key stages: detection and classification. Detection focuses on distinguishing between MRI images that either show a tumor (abnormal) or are tumor-free (normal). Classification is concerned with sorting glioma MRI images into either high-grade or low-grade gliomas^10^.

In radiology, using artificial intelligence (AI) can reduce error rates beyond what is achievable by human effort alone^11^. Machine learning (ML) and deep learning (DL), both are subsets of AI, assist radiologists in rapidly detecting and classifying tumors without the need for surgical procedures^12^. Deep learning techniques have recently drawn heightened interest in the research community. The Convolutional Neural Network (CNN) is a deep learning technique that has recently seen significant success in addressing medical imaging challenges^13^. CNNs have the capability to automatically extract essential features while reducing dimensionality^14,15^. This eliminates the need for traditional handcrafted features, as CNNs independently learn the critical features necessary for making final predictions^16^. Multiple CNN models have been utilized for brain tumor classification, showing remarkable success in medical classification tasks. These models have also been effective in areas such as skin lesion detection, breast cancer diagnosis, cardiac fibrosis detection, stroke identification, and arrhythmia classification^17–20^.

The high prevalence of brain tumors has led to the accumulation of extensive MRI data. Gliomas are particularly difficult to detect due to their irregular shapes and unclear boundaries. Detecting and classifying brain tumors are crucial processes that rely heavily on the expertise of diagnosing physicians. Despite these advancements, a significant gap remains in classifying low-contrast brain MRI images. This challenge is particularly evident when working with limited datasets^7^. The gap is two-fold. First, the lack of robust, adaptive enhancement for low-contrast MRI limits the performance of subsequent classifiers. Second, CNN-based approaches face inherent architectural limitations. Existing CNNs are optimized to learn global context and high-level shapes. However, their hierarchical pooling layers progressively reduce spatial resolution. Consequently, these networks are often less effective in capturing subtle, fine-grained textural patterns indicative of glioma grades. Furthermore, relying solely on deep features with limited data increases the risk of overfitting. Therefore, fusing deep semantic features from Transfer Learning CNNs (TRCNNs) with handcrafted texture descriptors is essential. This explicit fusion preserves diagnostically critical information and enhances model generalization^21,22^. Developing an intelligent system for contrast enhancement, detection, and classification of brain tumors is vital for assisting physicians. Such a system might serve as a significant resource in hospital emergency departments, enabling quicker diagnoses. For instance, such a technology might assist clinicians by analyzing images from an MRI to determine if they are normal or indicative of HG-G or LG-G. After an image is automatically detected as abnormal, clinicians may simply detect the brain tumor to assess its type during the classification stage. They can then identify the most suitable treatment approach for the severity of the brain tumor and manage it accordingly^23^.

To address these limitations and bridge the gap between deep semantic understanding and fine-grained texture analysis, this study introduces a hybrid framework. It begins with a specialized Adaptive Gamma Correction (AGC) and Denoising Convolutional Neural Network (DnCNN) preprocessing pipeline designed to simultaneously enhance contrast and suppress noise. Unlike standard approaches, our method introduces CNN-GLCM Fused Features (CGFFs), a new feature-level fusion strategy that explicitly combines deep features from three diverse TRCNNs with handcrafted texture descriptors to capture both global abstractions and micro-patterns. These composite feature vectors are subsequently utilized in a robust classification phase involving multiple classifiers: Support Vector Machine (SVM), Random Forest (RF), Extreme Gradient Boosting (XGBoost), Light Gradient Boosting Machine (LightGBM), and Stacked Ensemble model, which collectively aim to improve diagnostic reliability. The classifiers are trained to effectively discriminate among HG-G, LG-G, and normal brain MRI categories from BraTS 2020 dataset, demonstrating enhanced diagnostic precision. To further validate the reliability of these improvements, the statistical significance of the system’s performance is rigorously verified using the Friedman test. The proposed framework contributes significantly to the field through:

1. Development of MRI image enhancement strategy combining AGC and DnCNN techniques applied to the BraTs 2020 dataset, effectively addressing image contrast deficiencies and noise interference.
2. Development of a multi-feature fusion approach, integrating deep CNN layer features with GLCM-derived texture descriptors to form the robust CGFFs representation.
3. Comprehensive utilization of advanced classifiers as RF, XGBoost, LightGBM, SVM and a Stacked Ensemble, systematically trained and optimized to enhance overall classification robustness.
4. Demonstration of high computational efficiency with a total inference time of approximately 0.723s per image, validating the framework’s potential for integration into real-time clinical diagnostic workflows.
5. Implementation of Friedman test to statistically evaluate the performance of the five classifiers across the nine CGFFs representations.
6. Extensive validation of the proposed methodology against contemporary state-of-the-art techniques, demonstrating superior performance across critical metrics.

The paper is structured as follows: “Related work” section reviews relevant literature on brain tumor diagnosis. “Methodology” section introduces the proposed methodology. “Examination environment” section describes the experimental setup, datasets, and evaluation criteria. “Results and discussion” section presents and discusses the experimental findings comprehensively. Lastly, “Conclusion” section concludes the study.

## Related work

In recent years, significant research attention has been focused on detection of tumor presence or not and the classification of brain tumors. The accuracy of tumor diagnosis has been enhanced through the application of various automated detection and classification techniques. Machine learning and deep learning approaches play a vital role in the identification and categorization of brain tumors. For brain tumor detection targeting, Togacar et al.^24^ introduced a CNN model called BrainMRNet to detect normal and tumor MR images. They demonstrated that their model surpassed the performance of pretrained CNN models, achieving an accuracy of 96.05%. In^25^, a combination of five deep learning and five machine learning models were implemented to identify tumors in MRI scans. Each model was applied on 2156 MRI images. An ensemble algorithm, based on majority voting, was used to enhance the diagnostic accuracy of the models. Remarkably, the results achieved an accuracy of 96.51%. Alanazi et al.^26^ constructed a standalone CNN model with 22 layers for detecting the presence or absence of tumors. This model is then fine-tuned to adjust neuron weights for brain tumor detection through transfer learning. Consequently, the transfer-learned model achieves a high accuracy of 95.75%.

In^27^, various CNN architectures were utilized to create feature maps, which were then classified to identify possible tumor samples. The approach was tested on a brain dataset consisting of 253 MRI images, of which 155 contained tumors. The method achieved classification accuracies of 96% with CNN, 98.5% with VGG-16, and 98.14% with an ensemble model. Regarding the work in^28^, ResNet, AlexNet, and VGG were applied into data containing 253 MRI images. Due to the challenges posed by limited data availability in medical imaging, this study expanded the dataset through techniques such as flipping and rotation. Using the VGG-16 model architecture, the approach achieved a detection accuracy of 96%. Salama et al. introduced a system for brain tumor detection by creating more datasets using generative models. The dataset used in the study comprises 253 MRI images, with the generative model expanding it to a more robust size. They applied a classifier for reliable tumor detection in MRI images. The framework achieved a detection accuracy of 96.88%^29^.

In^30^, authors applied CNN-based pretrained models, specifically VGG-16, ResNet-50, and Inception-v3, to detect the presence of tumors. By leveraging transfer learning, they enhanced prediction accuracy while optimizing computational time. The VGG-16 model achieved the highest accuracy of 96%. In another work, six different machine learning algorithms were employed, including random forest, naïve bayes, neural network, CN2 rule induction, support vector machine, and decision tree, to enhance accuracy in addressing this gap. These methods were tested on a Kaggle dataset, where classification accuracy was assessed. A tenfold cross-validation approach was used to reinforce the training and testing processes. The findings reveal that SVM achieved the highest performance among the algorithms, with an accuracy of 95.3%^31^. In 2024, a hybrid detection framework was implemented to identify brain tumors. This system integrated five distinct CNNs for feature extraction, with each network selecting the best five layers for this purpose. These extracted features were then classified using SVM. They had an accuracy of 97.22%^32^.

Recently, a significant amount of research has been directed towards differentiating between HG-G and LG-G using various automated classification methods. Authors in^33^ explored CNN-based transfer learning for preoperative glioma grading on conventional MR images. Two CNN architectures AlexNet and GoogLeNet were evaluated, trained both from scratch and fine-tuned. Using fivefold cross-validation, the best model GoogLeNet with fine-tuning attained around 91% test accuracy. In^34^, the authors introduced a machine learning technique for differentiating LG-G from HG-G by analyzing radiomic features. This research employed MRI images from the MICCAI BRATs 2015 training set. After extracting features, the model classified tumor grades using ML algorithms, achieving an overall accuracy of approximately 91.3%. Mzoughi et al.^35^ proposed a deep multi-scale 3D CNN architecture using the BraTS 2018 dataset. Their model was trained in 284 volumetric MRI cases and validated in 67 cases. The 3D CNN employed small 3D kernels to capture local and global contextual features. This fully automatic method yielded 96.5% accuracy.

Ge et al. developed a semi-supervised learning framework for glioma grading that utilizes both labeled and unlabeled MRI data. They worked with the BraTS 2017 dataset containing 285 subjects across four MRI modalities. The process began by extracting deep CNN features and then utilizing a graph-based technique for label propagation. A constraint ensured consistency between 3 and 2D data to assign pseudo-labels to unlabeled scans. A CNN classifier was subsequently trained on the augmented labeled set, and GAN-generated synthetic MRI slices were added to combat overfitting on the moderate-sized dataset. This strategy achieved 90.7% test accuracy^36^. Özcan et al. introduced a custom CNN and compared it against pre-trained AlexNet, GoogLeNet, and SqueezeNet for classifying gliomas. The models were trained and evaluated on MRI scans from 104 patients. The approach used data augmentation and five-fold cross-validation to improve generalization. The custom CNN achieved the best results with an accuracy of 97%^13^. In^37^, the authors built a high-precision ensemble model using deep transfer learning and stacking. In this approach, seven distinct CNN architectures were fine-tuned using a brain tumor MRI dataset. The ensemble then integrated predictions from these models, selecting the result with the highest confidence as the ultimate decision. A tenfold cross-validation was used to evaluate performance. This model achieved accuracy around 98%.

Gull et al.^38^ proposed a CNN-based pipeline for classification, training on the BraTS 2020 dataset. Their model achieved an accuracy of 98.25%. Farajzadeh et al.^39^ introduced a deep hybrid representation learning approach that combines a CNN with traditional machine learning classifiers and evolutionary optimization. This hybrid model obtained an average classification accuracy of 98%. Ali et al.^40^ introduced a federated deep learning framework based on MRI data. Their method employed a 3D federated learning architecture trained on MRI scans from 142 patients obtained from the MICCAI dataset. The federated model utilized a multi-stream convolutional network combined with focal loss optimization to address class imbalance, achieving an accuracy of 90.72%. Nazir et al.^41^ propose a 3D CNN built on a U-Net with a multi scale feature attention module for glioma grading. Using the shared encoder’s feature vector and three dense layers with SoftMax, their classifier achieves 95.1% accuracy on the test set. Liu et al.^42^ conducted a comparative study evaluating Large Language Models (LLMs) against fine-tuned 3D CNNs for glioma classification on BraTS 2020. Their custom 3D CNN baseline achieved 80.0% accuracy on the binary classification of HGG vs. LGG.

Recent research has focused on automatic classification techniques used to categorize MRI brain images into normal, HG-G and LG-G. An automated framework for glioma classification was developed using a modified CNN architecture based on AlexNet^43^. This study focused on analyzing MRI scans obtained from the Cancer Imaging Archive (TCIA). The proposed model incorporated optimized convolutional and pooling layers to enhance feature extraction from MRI slices. The framework demonstrated a classification accuracy of 91.16% across three categories. An advanced cascaded deep convolutional neural network was designed to detect and classify gliomas^10^. The framework employed dual-path CNN architecture, enhanced with residual connections, to distinguish between normal, LG-G, and HG-G cases in MRI scans. The model was trained and evaluated using 1,800 MRI images from the BraTS 2017 dataset. It achieved an outstanding classification accuracy of 98.88%. Salama et al.^23^ developed the DCBT system, utilizing a transfer-learned EfficientNet-B0 backbone combined with a parallel CNN path for feature extraction. Evaluating their model on the BraTS 2020 dataset for a 3-class task (HG-G, LG-G, Normal), they achieved an accuracy of 98.5%. Topannavar et al.^44^ proposed a framework integrating Spatial Transformer Networks (STN) and Non-local Attention Mechanisms (NAM) into ResNet50 for 3-class classification (HG-G, LG-G, Normal) on BraTS 2020. Their approach achieved 98.66% accuracy. The various approaches presented have been summarized in Table 1, providing a comprehensive comparison of their techniques and performance metrics.

**Table 1:** A comparative analysis of various studies on glioma detection and classification.

Task	References	Year	Technique	Dataset	Accuracy (%)	Limitation
Detection (normal/tumor)	^24^	2020	BrainMRNet	Kaggle	96.05	Lacks interpretability, making predictions hard to understand
	^25^	2021	Ensemble CNN	Clinical MRI	97.80	Combining multiple models increases computational cost
	^26^	2022	Custom CNN + Transfer Learning	CE-MRI (Figshare)	95.75	Does not evaluate robustness under different noise conditions
	^27^	2022	CNNs + Ensemble	253 MRI	98.5	High accuracy on a small dataset poses a risk of overfitting
	^28^	2022	CNNs with Augmentation	253 MRI	96	Relies on basic augmentation techniques
	^29^	2022	Generative Model + CNN	253 MRI	96.88	Generative augmentation can add unrealistic patterns absent in real MRI scans
	^30^	2022	CNNs	–	96	Relies on standard transfer learning models without architectural changes
	^31^	2023	CNN + ML classfiers	Kaggle MRI	95.3	Handcrafted features often overlook complex patterns
	^32^	2024	CNN + SVM	394 MRI 120 MRI	97.22	Small dataset used for training and testing
Classification (HG-G/LG-G) (HG-G/LG-G/normal)	^33^	2024	GoogLeNet	Brain MRI	91	The performance is limited by a relatively small training set
	^34^	2025	ML on radiomic features	BraTS 2015	91.3	Handcrafted radiomic features and prior segmentation, limiting full utilization of imaging data
	^35^	2020	Deep 3D Multi-scale CNN	BraTS 2018	96.5	3D CNN models require high computational resources
	^13^	2021	Custom CNN, AlexNet, GoogLeNet	104 Patients MRI	97	Lacks comparison with more recent or advanced deep models
	^37^	2021	Ensemble of 7 CNNs + Stacking	Brain MRI	98	The design is resource-intensive and complex
	^38^	2022	CNN	BraTS 2020	98.25	Need for large volumes of labeled MRI data for training
	^39^	2023	Hybrid deep CNN + ML	BraTS 2020	98	High computational cost
	^40^	2023	Federated multi-stream 3D CNN	BraTS 2017	90.72	The design indicates challenges in maintaining performance consistency across distributed data
	^41^	2024	3D CNN with U-Net	BraTS 2020	95.1	High computational and memory requirements
	^42^	2025	3D CNN and LLM	BraTS 2020	80	Limited classification accuracy
	^43^	2024	Modified AlexNet	TCIA MRI	91.16	Small dataset used for training and testing
	^10^	2024	Cascaded CNN with Residual Paths	BraTS 2017 (1800 MRIs)	98.88	The design is resource-intensive and complex
	^23^	2025	DCBT	BraTS 2020	98.5	lacking the feature diversity
	^44^	2025	ResNet50 + STN-NAM	BraTS 2020	98.66	High computational complexity

In summary, previous studies encountered common challenges (as outlined in Table 1), including limited explanation capability for predictions, susceptibility to image noise, overfitting due to small training datasets, extensive computational demands, dependence on manually designed features, and inadequate representation of image textures. The proposed model in this study introduces an advanced method specifically developed to solve these drawbacks. Initially, the AGC-DnCNN preprocessing approach is applied, significantly enhancing MRI images by improving their contrast and reducing noise, thereby ensuring reliable results across various imaging conditions. Subsequently, the framework introduces CGFFs, effectively merging deep semantic features obtained from CNN models with detailed texture descriptors derived from GLCM, addressing previous shortcomings related to texture representation. Additionally, the utilization of multiple TRCNNs, each optimized independently with different optimization algorithms, diversifies the extracted features, reducing the risk of overfitting, particularly valuable when dealing with small datasets. This comprehensive strategy resolves critical issues highlighted in previous studies and establishes a powerful and precise system for brain tumor classification, demonstrating clear improvements compared to previous approaches.

## Methodology

**Fig. 1:**
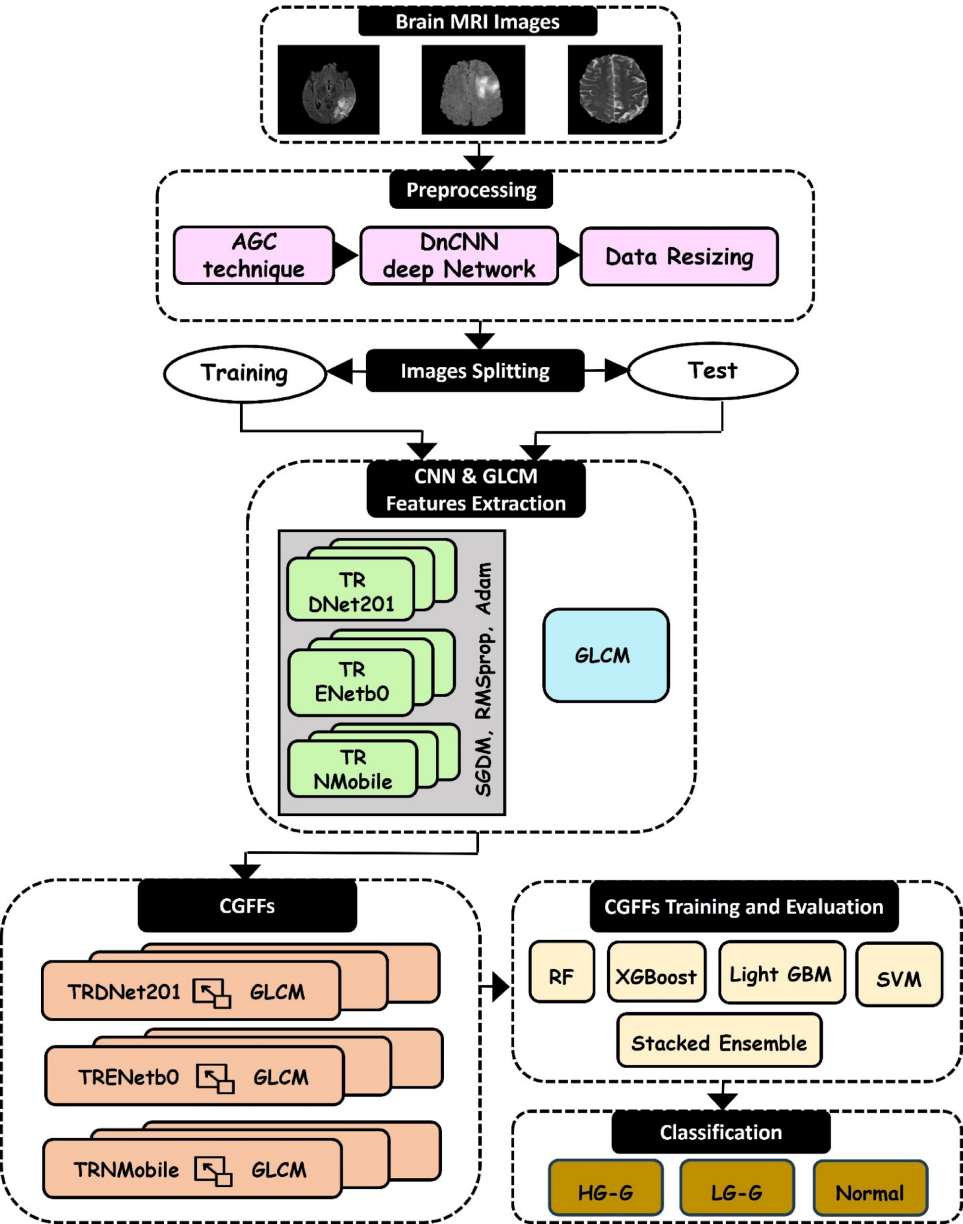
The overall structure of the proposed model comprises three primary phases: an initial preprocessing phase including AGC and DnCNN models, feature extraction through two pathways leading to the fused layers (CGFFs), and evaluation phase utilizing various classifiers.

The proposed system shown in Fig. 1 initiates with preprocessing brain MRI images using a hybrid enhancement pipeline that integrates AGC and DnCNN, followed by uniform resizing to standardize input dimensions. The preprocessed dataset is then divided into training and testing subsets to facilitate supervised learning. Feature extraction is performed using three TRCNN models: TRDNet201, TRENetB0, and TRNMobile. For each architecture, transfer learning is applied by replacing the original final classification layer to support the three target classes. Additionally, each model is individually fine-tuned through distinct optimizers: SGDM, RMSprop, and Adam to optimize feature representation. From each TRCNN, the three most informative layers are selected, and their deep features are concatenated with texture features extracted via GLCM, forming a total of nine CGFF representations. This fusion effectively captures semantic and texture-based characteristics within the MRI data. The resulting CGFF vectors are subsequently input to multiple classifiers; RF, XGBoost, LightGBM, SVM and a Stacked Ensemble to enhance classification robustness. The framework is designed to accurately differentiate between HG-G, LG-G, and normal cases.

### Brain images preprocessing

**Fig. 2:**
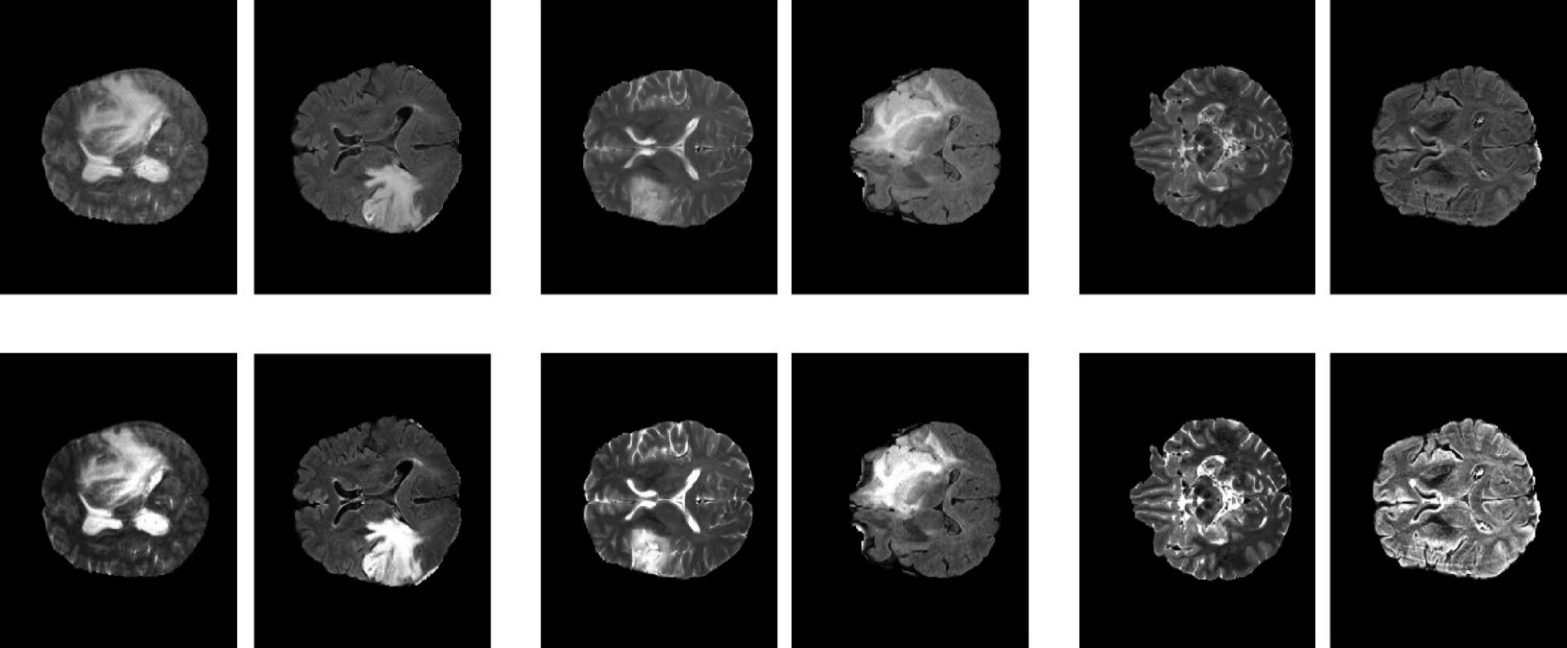
The output of the preprocessed three-class images. The first two columns from the left contain HG-G images, the next two columns contain LG-G images, and the final two columns contain normal images. Original images are displayed in the top row, while preprocessed images are shown in the bottom row.

Image enhancement and noise reduction are pivotal techniques in medical image processing, crucial for improving image quality and ensuring accurate diagnosis. The primary goals of these techniques are to enhance contrast, highlight important features, suppress irrelevant details and improve the performance of automated systems for detection and classification. In our study, we utilize MRI brain images obtained from the BRATS 2020 dataset, specifically T2-weighted and FLAIR modalities, which include labeled data for three diagnostic categories HG-G, LG-G and normal. To ensure optimal image clarity, the proposed framework employs a specific sequential pipeline: AGC followed by DnCNN. This sequence is designed to first enhance global contrast and reveal fine structural details, enabling the subsequent DnCNN stage to effectively preserve these features while suppressing noise. Theoretically, applying denoising prior to contrast enhancement risks amplifying residual noise artifacts during the contrast adjustment phase. Fig. 2 illustrates the proposed preprocessing algorithm for all diagnosis classes.

### The AGC

AGC is an image processing technique designed to enhance the visual quality of an image by adjusting its brightness and contrast. Gamma correction involves modifying the luminance of an image through a non-linear transformation controlled by a gamma value (γ). While standard adaptive methods calculate γ locally, such approaches often amplify background noise in low-contrast MRI slices. To address this, this study adopts a dataset-optimized global approach. The γ value was empirically determined through a comprehensive grid search (ranging from 0.5 to 2.5) tailored to the BraTS 2020 dataset characteristics, selecting γ = 1.65 as the optimal parameter that maximizes tumor contrast without noise amplification. The transformation is governed by Eq. (1):

1 $$P_{out} = P_{in}^{\gamma }$$

where $P_{in}$ is the input pixel intensity, γ is the gamma value and $P_{out}$ is the output pixel intensity. This optimized transformation adjusts pixel values to make anatomical details more discernible, providing a stable and noise-resistant enhancement suitable for the subsequent denoising stage.

### The DnCNN

DnCNN is a deep convolutional neural network that aims to remove noise from images while preserving important details. The network is trained in a supervised manner on pairs of noisy and clean images to learn the mapping between noisy inputs and clean outputs. DnCNN takes a noisy image as input and processes it through its layers to generate a denoised output. The network leverages its learned filters to suppress noise while preserving essential features and structures in the image. The depth of the network and the size of convolutional (Conv) kernels are typically chosen based on the complexity of the denoising task and the available computational resources. The DnCNN model comprises 59 layers arranged sequentially, including an input layer, one layer block of Conv + Relu, 18-layer blocks of Conv + batch normalization (BN) + ReLU, one layer of Conv and the final layer is a regression layer that outputs the residual noise. The denoised image is obtained by subtracting the network’s output from the noisy input. Formally, let $x$ denote the noisy input image and $R\left( x \right)$ represent the predicted residual noise estimated by the DnCNN. Consequently, the final denoised output image $\hat{y}$ is defined in Eq. (2) as:

2 $$\hat{y} = x - R\left( x \right).$$

**Fig. 3:**
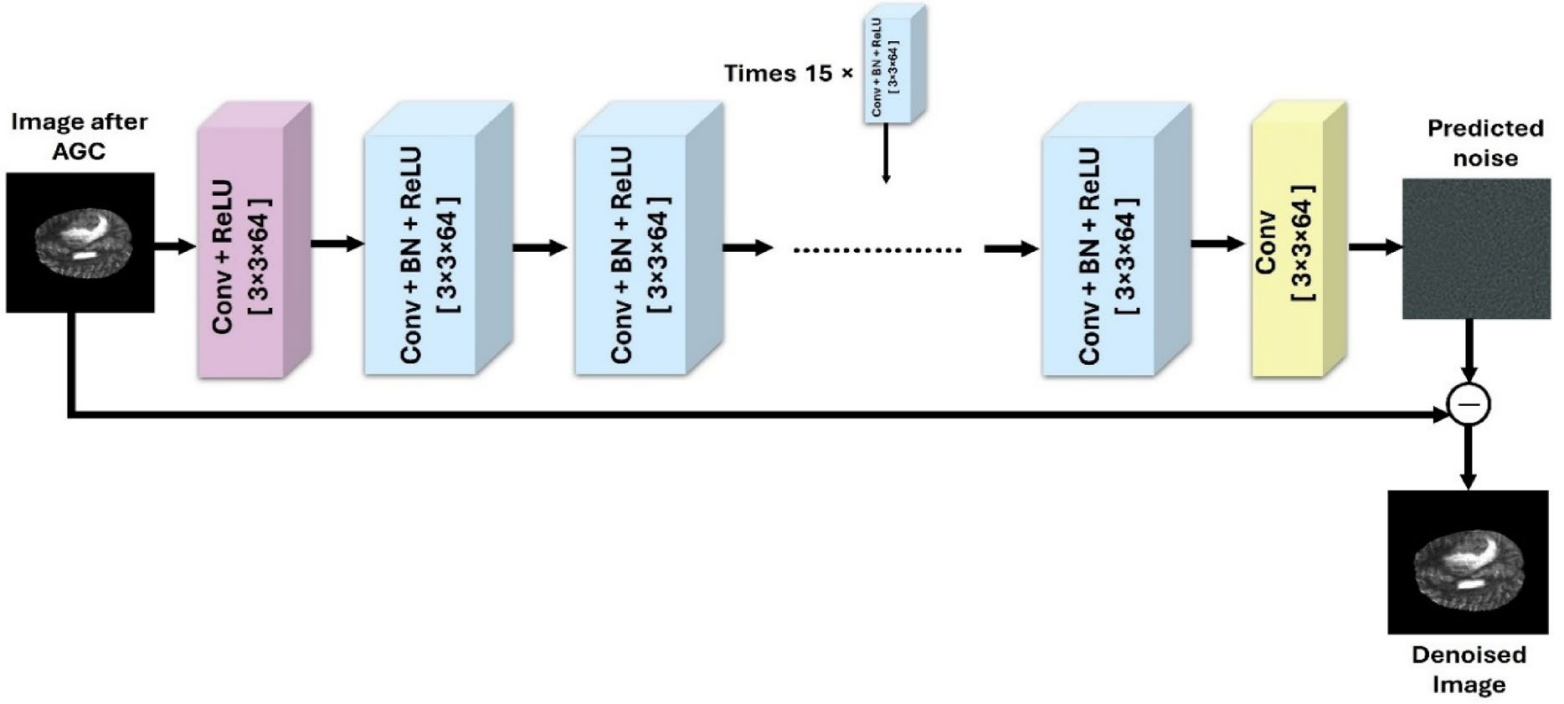
The structure of the DnCNN network.

The network is trained to minimize the Mean Squared Error (MSE) between the predicted residual noise and the true residual noise. Fig. 3 shows the architecture of DnCNN, where three of the Conv + BN + ReLU blocks are explicitly drawn, while the remaining 15 are denoted using a repetition marker.

### Data resizing for CNNs

Resizing the images is necessary to ensure they are compatible with the employed CNN networks. The first network is DenseNet201; it has 201 deep layers including dense blocks and transition layers, allowing it to learn complex and hierarchical features^45^. As detailed by MathWorks, the complete network includes 708 operational layers from the input to the classification stage, with approximately 20 million learnable parameters^46^. The second Network is EfficientNetB0, according to MathWorks, it has a total of 290 layers with size of 5.3 million. This architecture is part of the EfficientNet family, known for its balanced approach to model scaling that optimizes accuracy and computational efficiency^46^. The last one is NmasNetMobile network which consists of repeated building blocks, enabling a highly efficient and modular structure. Despite being designed for mobile environments, NasNetMobile achieves high accuracy in image classification. NasNetMobile comprises 913 layers with a size of 5.3 million, representing the total learnable parameters. This work employs a dataset comprising 2100 images, each originally sized at 240 × 240 pixels. To align with the input dimension requirements of DenseNet201, EfficientNetB0, and NASNetMobile, all images are uniformly resized to 224 × 224 pixels.

### Fusion of multi-level feature extraction (CGFFs)

Feature extraction plays a crucial role in brain tumor classification by leveraging both deep learning and texture-based methods. In this work, two main types of features are extracted: deep features from CNNs and handcrafted texture features using the GLCM. For the deep features, various layers from TRDNet201, TRENetB0, and TRNMobile are employed. To identify the most informative layers, a comprehensive empirical evaluation was conducted. We extracted features from various depths within each CNN architecture and evaluated their individual classification performance. The top three layers were selected because they consistently yielded the highest accuracy and class separability. These layers are strategically located at different stages of the network to capture a rich hierarchy of features, ranging from fine-grained textural details to high-level semantic abstractions. To further optimize feature extraction, SGDM, RMSprop, and ADAM optimizers were employed during the training process. These optimizers adjust the CNN weights based on the gradient of the loss function. A comparative analysis was performed to determine the most suitable optimizer for enhancing deep feature quality. Table 2 outlines the selected layers, their positions within the networks, and the extracted activations.

**Table 2:** The parameters of the optimal three layers from each network.

Network	Layer order	Layer type	Activations
TRDNet201	165	ReLU	[14, 14, 128]
	493	Depth concatenation	[7, 7, 960]
	704	Batch normalization	[7, 7, 1920]
TRENetB0	115	2-D convolution	[1, 20]
	178	Batch normalization	[14, 14, 672]
	273	Element-wise multiplication	[7, 7, 1152]
TRNMobile	748	Batch normalization	[7, 7, 176]
	895	Batch normalization	[7, 7, 176]
	905	Addition	[7, 7, 176]

The second type of feature extraction involves GLCM, which provides valuable texture information by analyzing the spatial relationship between pixel intensities. As a second-order statistical method, GLCM captures structural characteristics such as contrast, correlation, energy, and homogeneity. These features are particularly effective in highlighting subtle textural patterns relevant to glioma classification^47^. In this study, the GLCM calculation was performed using a pixel distance of d = 1 and an angle of θ = 0 degree. Consequently, ten statistical features were computed from the GLCM as defined in Table 3. While deep learning models offer strong capabilities in capturing abstract and high-level semantic features, they often lack sensitivity to fine-grained texture patterns that are essential in distinguishing subtle tumor characteristics. To address this limitation, a feature-level fusion strategy is proposed by integrating CNN-derived deep features with handcrafted texture descriptors obtained via the GLCM.

**Table 3:** Statistical extracted features from GLCM.

Feature No	Feature name	Equation
1	Contrast	$\mathop \sum \limits_{i,j}^{n - 1} \left| {i - j} \right|^{2} { }f\left( {i,j} \right)$
2	Correlation	$\mathop \sum \limits_{i,j}^{{n - 1{ }}} \frac{{\left( {i - \mu_{x} } \right)\left( {j - \mu_{y} } \right)f\left( {i,j} \right)}}{{\sigma_{i} \sigma_{j} }}$
3	Energy	$\mathop \sum \limits_{i,j}^{n - 1} f\left( {i,j} \right)^{2}$
4	Homogeneity	$\mathop \sum \limits_{i,j}^{n - 1} \frac{{f\left( {i,j} \right)}}{{1 - \left( {i - j} \right)^{2} }}$
5	Dissimilarity	$\mathop \sum \limits_{i,j} \left| {i - j} \right|.f\left( {i,j} \right)$
6	Entropy	$\mathop \sum \limits_{i,j} f\left( {i,j} \right)\log \left( {f\left( {i,j} \right)} \right)$
7	Difference variance	$variance{ }of{ }f_{x - y}$
8	Maximum probability	$\mathop {\max }\limits_{i,j} f\left( {i,j} \right)$
9	Inverse difference moment	$\mathop \sum \limits_{i = 1}^{n} \mathop \sum \limits_{j = 1}^{n} \frac{{f\left( {i,j} \right)}}{{1 + \left| {i - j} \right|^{2} }}$
10	Inverse difference moment normalized	$\mathop \sum \limits_{i = 1}^{n} \mathop \sum \limits_{j = 1}^{n} \frac{{f\left( {i,j} \right)}}{{1 + \left( {i - j} \right)^{2} /n^{2} }}$

Regarding the feature fusion process, each selected CNN layer’s deep feature vector is fused explicitly with texture descriptors extracted from GLCM features. This fusion procedure combines the high-level semantic insights provided by CNN features with the fine-grained textural characteristics identified by GLCM, yielding nine enriched, unified feature representation termed CGFFs. Specifically, each of the top three layers selected from TRDNet201, TRENetB0, and TRNMobile provides a 3D feature map. To ensure compatibility with the 1D texture descriptors, these 3D maps are processed using a flattening operation. This converts the 3D tensors into 1D feature vectors, thereby preserving the complete spatial information extracted by the CNNs. These vectors are then individually fused with the corresponding GLCM feature vector. Regarding the feature fusion dimensionality, the final CGFF vector size is the sum of the flattened deep feature dimension and the GLCM feature dimension. This fusion is designed to enrich the descriptive power of the feature space, allowing the model to simultaneously capture both global patterns and micro-structural variations that may be diagnostically relevant in differentiating glioma classes.

### Classification techniques

Following the fusion stage, the resulting nine CGFFs features were evaluated using five supervised machine learning classifiers to categorize brain MRI scans into HG-G, LG-G, and normal classes. The selected classifiers include RF, XGBoost, LightGBM, SVM and a Stacked Ensemble model that combines the strengths of individual learners.

Random Forest is an ensemble-based classification algorithm that builds multiple decision trees during training^48^. Each tree is trained on a bootstrapped subset of data, with random feature selection at each split, ensuring model diversity. Final predictions are made through majority voting across all trees^48^. RF is highly effective in handling high-dimensional datasets and is tolerant of missing values. It also provides feature-important metrics, which help identify the most influential variables^49^. Although excessive similarity among trees can reduce classification accuracy, RF remains a strong, interpretable, and efficient model for multi-class medical image classification.

Extreme Gradient Boosting is an efficient gradient boosting algorithm known for its high accuracy and scalability. It builds decision trees sequentially, where each new tree minimizes the residual errors of prior ones using a second order Taylor approximation of the loss function^50^. XGBoost avoids overfitting and performs reliably in complex tasks such as classifying using combined features. Its support for parallel computing, handling missing values, and sparsity aware algorithms enhances its performance in clinical datasets^50^. In the proposed system, XGBoost effectively learned the non-linear relationships embedded in hybrid features, allowing precise differentiation between HG-G, LG-G, and normal brain images.

Light Gradient Boosting Machine is a gradient boosting framework optimized for speed and efficiency on high dimensional data. It employs a leaf-wise tree growth strategy along with histogram-based feature binning, which helps speed up training and allows for deeper trees than traditional methods. LightGBM natively handles categorical features and supports parallel learning, making it ideal for real-time medical applications. In this study, LightGBM efficiently processed concatenated CNN–GLCM features, providing accurate multi-class classification across brain tumor grades. Its capacity to reduce memory consumption and adapt to imbalanced data makes it a valuable component in the classification pipeline of glioma detection using MRI slices.

Support Vector Machine is a widely used supervised learning algorithm for classification tasks. It works by finding the optimal hyperplane that separates data points of different classes with the maximum margin^51^. While SVM is inherently a binary classifier, it can be extended to handle multi-class classification problems, such as distinguishing between three classes. Error-Correcting Output Codes (ECOC) extend the capability of SVM for multi-class classification by encoding each class into a unique binary string, or code. For a three-class problem, ECOC generates binary codes for each class and decomposes the multi-class problem into multiple binary classification tasks^51^. Each binary classifier is trained to distinguish between two subsets of the classes, as defined by the binary codes. For instance, considering three classes designated X, Y, and Z, they can be encoded as follow: 00, 01 and 10.

The Stacked Ensemble model is critical to our framework, designed to synthesize the predictions of the base classifiers (RF, XGBoost, LightGBM, and SVM) by training a meta-classifier on their outputs. In this study, a Logistic Regression model was employed as the meta-classifier due to its interpretability and efficiency in combining probabilistic outputs from heterogeneous base learners. This hierarchical structure aims to reduce prediction variance by exploiting the diverse predictive power of its base learners. Its true advantage in this application lies in integrating fundamentally different classification paradigms. For instance, the SVM is highly adept at finding an optimal separating hyperplane in the high-dimensional CGFF feature space. In contrast, the tree-based models (RF, XGBoost, and LightGBM) excel at capturing complex, non-linear, and hierarchical interactions between features by creating a series of axis-parallel decision boundaries. By synthesizing these complementary strengths, the meta-learner intelligently weights each model’s contribution, improving the detection of subtle differences between tumor types. This mitigates the risk of overfitting to the biases of any single model and achieves a more generalized and robust final classification, ensuring the high reliability and adaptability required for a clinical diagnosis^52,53^.

## Examination environment

### Machine tool

The work was conducted on a device with the following specifications: an AMD Ryzen 7 5800H processor with Radeon Graphics, CPU running at 3.20 GHz, 16.0 GB of RAM, NVIDIA GeForce RTX 3070 with 8 GB and a 64-bit operating system × 64-based processor. The computations for the proposed system were implemented using MATLAB R2024a.

### Dataset overview

The dataset used in this study is derived from the BraTS 2020 collection^54^, which natively consists of 3D multimodal MRI volumes. In this work, we utilized the T2-weighted and Fluid- FLAIR modalities. To ensure compatibility with the proposed 2D CNN architectures, a 3D-to-2D conversion process was applied. Slices containing tumor regions were selected for the HG-G and LG-G classes, while slices from non-tumorous regions were extracted to form the Normal class. The final curated dataset comprises 2,100 2D images, originally sized at 240 × 240 pixels, which were subsequently resized to 224 × 224 pixels during preprocessing (Table 4).

**Table 4:** Training and testing dataset.

Training data			Testing data			Total dataset
HG-G	LG-G	Normal	HG-G	LG-G	Normal	2100
540	537	498	184	181	160	

### Evaluation criteria

The efficacy of the proposed method was assessed through two distinct criteria: the enhancement of the brain image and the classification of brain tumors. The enhancement outcomes were evaluated based on two factors: peak signal-to-noise ratio (PSNR) and structural similarity index measure (SSIM). The classification outcomes were appraised using five metrics: accuracy (AC), recall (RE), specificity (SP), positive predictive value (PPV) and negative predictive value (NPV) measures.

### Evaluating parameters for preprocessed brain images

The performance of the enhancement results was evaluated by measuring two key parameters: the peak signal-to-noise ratio and the structural similarity index measure. These parameters have been utilized across other multiple image enhancement techniques, specifically Histogram Equalization (HE), Adaptive Histogram Equalization (AHE), and Contrast Limited Adaptive Histogram Equalization (CLAHE).

The peak signal-to-noise ratio of a brain MRI image is a quality metric that compares the peak signal level to the amount of noise in the image. In the context of MRI imaging, the PSNR is especially helpful for assessing the fidelity of reconstructed or processed images since it quantifies the ratio of the greatest achievable signal power to the power of the image’s corrupting noise^55^. The formula for calculating the PSNR is denoted by Eq. (3):

3 $$PSNR = 10log_{10} \left( {\frac{PS}{{MSE}}} \right)$$

where the Peak Signal (PS) is the highest possible signal value, while the mean squared error (MSE) is a measure of the average squared difference between the original and processed image pixel values. A higher PSNR value implies greater picture quality, indicating that the image has fewer distortions and more original data content. A high PSNR in brain MRI imaging is desired because it indicates a better and more accurate picture of brain structures, allowing healthcare providers and researchers to make precise diagnoses and analyses^56^.

The structural similarity index measure is a statistic that measures the similarity of two images based on more than just their pixel values^55^. In the context of brain MRI imaging, SSIM is critical for assessing image quality and fidelity by quantifying brightness, contrast, and structure. The SSIM measure is useful for comparing the anatomical features contained in various brain scans, which aids in activities such as image registration and quality evaluation^56^. Equation (4) represents the formula used to compute SSIM.

4 $$SSIM\left( {f,g} \right) = \frac{{\left( {2\mu_{f} \mu_{g} + C_{1} } \right)\left( {2\sigma_{fg} + C_{2} } \right)}}{{\left( {\mu_{f}^{2} + \mu_{g}^{2} + C_{1} } \right)\left( {\sigma_{f}^{2} + \sigma_{g}^{2} + C_{2} } \right)}}$$

In this equation, $\mu_{f} \;and\;\mu_{g}$ represent the mean pixel values of the two images, $\sigma_{f}^{2} \;and\;\sigma_{g}^{2}$ denote their variances, and $\sigma_{fg}$ shows the covariance between the images. $C_{1}$ and $C_{2}$ are constants to prevent division by zero. The SSIM value ranges from − 1 to 1, with values closer to 1 signifying higher structural similarity between the images.

### Evaluating parameters for brain tumor classification

We evaluated classification performance using a combination of training and testing datasets. The evaluation of the classification model involved the use of the following parameters: AC (Eq. 5), which is a metric that measures the number of correct predictions based on both true positives and true negatives compared to the total number of predictions; RE (Eq. 6), which measures the number of cases that were positive accurately detected by the model; SP (Eq. 7), which measures the number of cases that were negative accurately detected by the model; PPV (Eq. 8), which is the fraction of true positives among all positive predictions; and NPV (Eq. 9), which is fraction of true negatives among all negative predictions.

5 $$AC = \frac{{\checkmark }P + {\checkmark }N}{{ \times P + \times N}}$$

6 $$RE = \frac{{\checkmark }P}{{{\checkmark }P + \times N}}$$

7 $$SP = \frac{{\checkmark }N}{{{\checkmark }N + \times P}}$$

8 $$PPV = \frac{{\checkmark }P}{{{\checkmark }P + \times N}}$$

9 $$NPV = \frac{{\checkmark }N}{{{\checkmark }N + \times P}}$$

where ${\checkmark }P$ is the number of true positives, ${\checkmark }N$ is the number of true negatives, $\times P$ is the number of false positives, and $\times N$ is the number of false negatives.

## Results and discussion

### Analysis of enhancement technique of preprocessed brain images

This section presents a two-stage analysis of the enhancement process applied to the BraTS 2020 dataset using PSNR and SSIM metrics. Initially, the performance of the proposed enhancement system (AGC followed by DnCNN) was compared against conventional enhancement methods, including HE, AHE and CLAHE. As illustrated in Table 5, the proposed method consistently outperformed traditional techniques across all image classes, achieving the highest PSNR and SSIM values. This result emphasized the potential of the proposed system to improve medical image clarity and enhance visibility of anatomical structures, especially in glioma detection.

**Table 5:** The evaluation of the performance of various image enhancement techniques.

Dataset	HE		AHE		CLAHE		Proposed method	
	PSNR	SSIM	PSNR	SSIM	PSNR	SSIM	PSNR	SSIM
HG-G	20.37	0.02	22.51	0.44	23.64	0.51	**25.98**	**0.97**
LG-G	20.25	0.02	24.59	0.44	25.77	0.51	**27.98**	**0.96**
Normal	18.32	0.01	18.82	0.39	20.61	0.45	**21.64**	**0.84**

Significant values are in [bold].

Subsequently, an ablation study was conducted to assess the impact of the enhancement order. Four combinations were tested: AGC only, DnCNN only, DnCNN followed by AGC (Dn-AGC), and AGC followed by DnCNN (AGC-Dn). As shown in Table 6, the results validated that applying AGC prior to DnCNN yielded the most favorable outcome. The rationale is that AGC enhances image contrast and reveals finer details before denoising is applied, allowing DnCNN to preserve the enhanced features while effectively removing noise. Conversely, applying DnCNN before AGC may lead to residual noise amplification during contrast enhancement. These observations confirmed the strategic importance of the processing order.

**Table 6:** Assessment of enhancement sequences based on PSNR and SSIM.

Enhancement technique	Class	PSNR	SSIM
AGC	HG-G	24.21	0.88
	LG-G	25.64	0.86
	Normal	20.90	0.80
DnCNN	HG-G	24.87	0.90
	LG-G	26.45	0.88
	Normal	21.12	0.82
Dn-AGC	HG-G	25.56	0.93
	LG-G	27.32	0.91
	Normal	21.45	0.83
AGC-Dn (proposed)	HG-G	**25.98**	**0.97**
	LG-G	**27.98**	**0.96**
	Normal	**21.64**	**0.84**

Significant values are in [bold].

### Evaluation of classification performance using various classifiers

Table 7 presents a detailed comparative analysis of the five advanced classifiers which were applied to the nine CGFFs sets of the three transferred CNNs. The classification performance was evaluated across five metrics: AC, RE, SP, PPV, NPV. Most of the results were obtained using the ADAM optimizer, as it consistently provided the best performance among the three tested optimizers. However, the values marked with (*) indicate cases where the RMSprop optimizer achieves superior results for those specific feature sets. A critical observation from the table is the impact of optimizer selection on the classification results. ADAM provided consistent and effective training for extracting deep features. However, in some layers, RMSprop led to better feature learning and improved the overall classification results. This hybrid optimization strategy underlines the importance of tailored model tuning in medical image analysis tasks, where minor improvements can substantially affect clinical utility.

**Table 7:** Comparative classification results for each classifier across the nine CGFFs layers.

Classifier	Metrics%	CGFFs of TRDNet201			CGFFs of TRENetB0			CGFFs of TRNMobile		
		165	493	704	115	178	273	748	895	905
RF	AC	92.95	94.10	92.76	**94.67**	94.48	94.10	93.14	88.38	91.62
	RE	94.42	95.12	94.29	**94.98**	94.82	94.86	93.66	90.37	92.44
	SP	96.76	97.24	96.66	**97.45**	97.38	97.24	96.84	94.98	96.22
	PPV	93.19	94.29	92.95	**94.85**	94.66	94.29	93.37	88.77	91.90
	NPV	96.38	96.98	96.29	**97.34**	97.34	97.0	96.60	94.15	95.92
XGBoost	AC	90.85	83.80	92.57*	94.28	**95.23**	94.09*	91.61	91.04*	89.33
	RE	92.97	87.28	93.54*	94.53	**95.36**	94.54*	92.48	91.24*	90.28
	SP	95.80	92.94	96.57*	97.23	**97.67**	97.13*	96.19	95.59*	95.17
	PPV	91.02	84.03	92.82*	94.44	**95.41**	94.20*	91.90	91.15*	89.59
	NPV	95.31	91.71	96.23*	97.15	**97.70**	96.99*	95.94	95.49*	94.81
LightGBM	AC	90.47	91.80*	86.66	92.38*	**96.76**	92.38*	94.28	91.42*	85.71*
	RE	92.54	92.58*	89.64	92.57*	**96.77**	92.57*	94.22	91.59*	88.11*
	SP	95.62	96.08*	93.80	96.33*	**98.40**	96.33*	97.21	95.75*	94.03*
	PPV	90.60	92.10*	86.84	92.47*	**96.88**	92.47*	94.26	91.46*	86.19*
	NPV	95.13	95.83*	93.21	96.19*	**98.44**	96.19*	97.16	95.68*	92.88*
SVM	AC	94.67	97.71	93.90	**99.05**	97.33	95.62	95.43	92.95	95.05
	RE	95.60	97.96	94.97	**98.99**	97.41	95.79	95.65	93.42	95.24
	SP	97.49	98.88	97.15	**99.52**	98.67	97.91	97.81	96.72	97.66
	PPV	94.84	97.77	94.08	**99.08**	97.43	95.76	95.56	93.14	95.21
	NPV	97.26	98.83	96.78	**99.54**	98.66	97.73	97.71	96.47	97.57
Stacked ensemble	AC	95.42*	95.42	92.57	93.90*	96	**96**	94.28	90.09*	85.90*
	RE	95.57*	96.09	94.09	94.24*	96.07	**96.28**	94.60	90.30*	88.30*
	SP	97.81*	97.82	96.59	97.11*	98.05	**98.08**	97.33	95.14*	94.10*
	PPV	95.58*	95.56	92.79	94.11*	96.14	**96.13**	94.48	90.21*	86.37*
	NPV	97.76*	97.66	96.19	96.95*	98.03	**97.99**	97.22	95.01*	92.96*

Significant values are in [bold].

Among the evaluated classifiers, it can be ranked from strongest to weakest as follows: SVM, LightGBM, Stacked Ensemble, XGBoost, and RF. SVM showed the highest and most consistent performance, particularly when applied to CGFF115 TRENetB0. This configuration recorded a remarkable accuracy of 99.05%, along with outstanding recall of 98.99%, specificity of 99.52%, and predictive values: PPV of 99.08% and NPV of 99.54%. The superior results from this CGFF stem from the architectural efficiency of TRENetB0, which balances network depth, width, and resolution. This structure enables CGFF 115 to extract mid-level semantic features that are both expressive and computationally efficient. When combined with SVM’s capacity to identify optimal decision boundaries in complex feature spaces, this led to highly accurate classification across the three MRI categories.

For LightGBM, its highest performance is attained with CGFFs 178 from TRENetB0, where it records an accuracy of 96.76%, recall of 96.77%, and specificity of 98.40%. The success of this configuration further validated the importance of layer 178 from TRENetB0 in producing highly discriminative features across various classifiers. Regarding Stacked Ensemble, the optimal performance appeared when using CGFFs of layer 178 from TRENetB0, achieving an accuracy of 96%, a recall of 96.07%, and a specificity of 98.05%. This CGFFs layer corresponds to a batch normalization stage that contributes to feature consistency for enhancing ensemble learning stability. The architectural strengths of TRENetB0 thus played a pivotal role in its repeated effectiveness across different classifiers. Notably, the single SVM outperformed the Stacked Ensemble, attributed to its superior capability in finding optimal separating margins within the high-dimensional CGFF feature space.

**Fig. 4:**
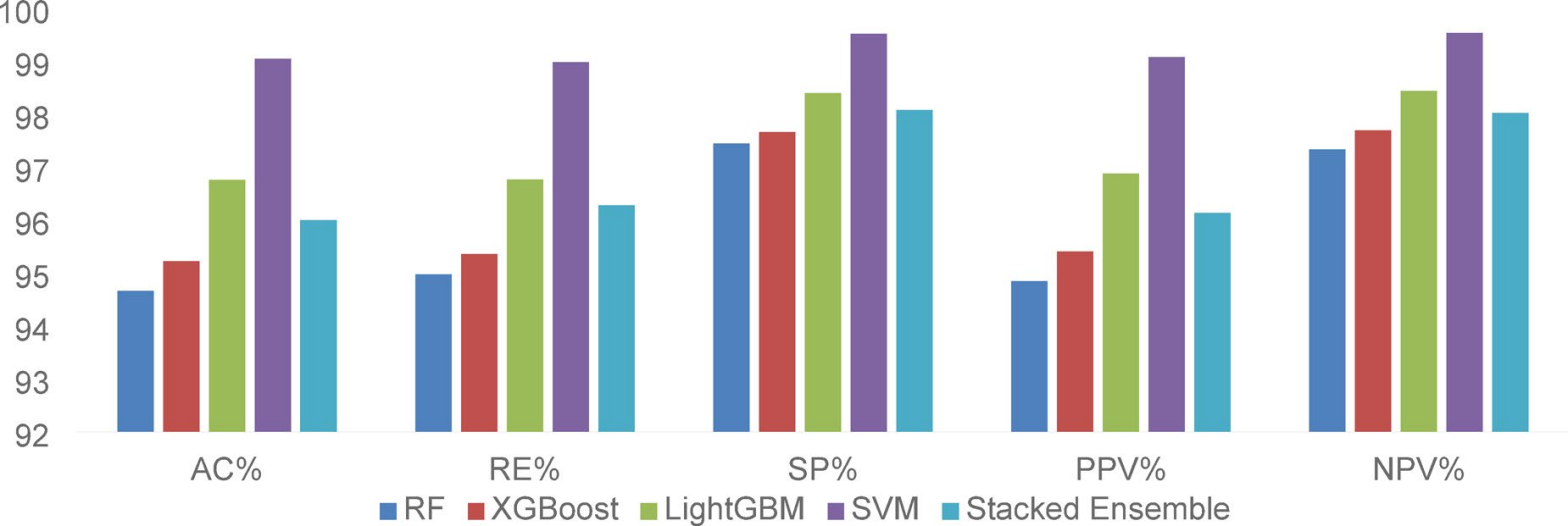
Comparative performance of classifiers based on evaluation metrics.

**Fig. 5:**
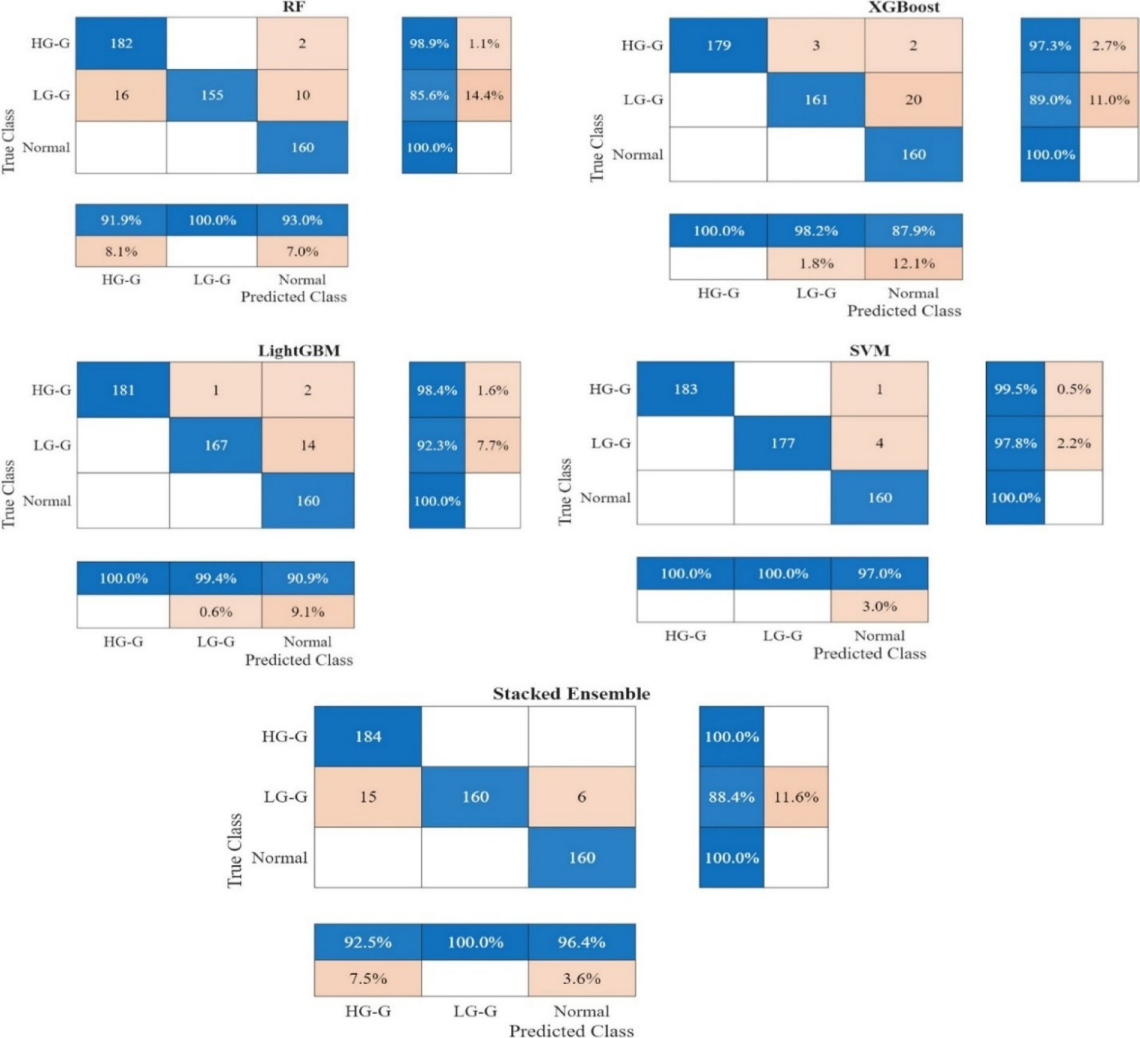
Heatmap visualization of the confusion matrix for the optimal performance for each classifier.

For XGBoost, the most favorable results emerged from CGFFs 178 from TRENetB0, with accuracy reaching 95.23%, recall at 95.36%, and specificity at 97.67%. RF performs best when utilizing CGFFs 115 from EfficientNetB0, achieving an accuracy of 94.67%, recall of 94.98%, and specificity of 97.45%. These differences are illustrated in Fig. 4 with the bar chart, where SVM’s metrics are highest across all five measures and the other classifiers exhibited progressively lower yet still high-performance levels. Fig. 5 illustrates the confusion matrix heatmap representing the optimal performance for each classifier. It was clearly observed that SVM achieved the best performance, demonstrating significantly fewer misclassifications compared to the other classifiers.

### Statistical analysis based on Friedman test

A Friedman test was applied to compare the performance of the five classifiers across the nine CGFF feature representations. This test is non-parametric, making it particularly suitable for repeated measures designs where the same data is evaluated across the classifiers^57^. The Friedman analysis revealed highly significant differences among the five classifiers for every evaluation metric. As summarized in Table 8, the Friedman test results indicated significant differences in classifier performance for all evaluated metrics. In particular, the null hypothesis of equal performance was rejected for AC, RE, SP, PPV, and NPV, with all *p*-values ≤ 0.002. All these values were well below the 0.05 significance level, confirming that at least one classifier outperformed the others in each metric. Any metric exceeding this value would indicate no significant statistical differences among classifiers, but this was not observed in the current analysis. Table 8 summarizes the specific Friedman test statistics, including the Chi-square $\left( {X^{2} } \right)$ values and corresponding *p*-values, confirming statistically significant performance differences across all evaluation metrics.

**Table 8:** The Friedman test results (Chi-square and *p*-value) for each evaluation metric, calculated with degrees of freedom $\left( {df} \right) = 4$.

Parameter	AC	RE	SP	PPV	NPV
Chi-Square ($X^{2}$)	17.64	19.37	18.4	17.51	17.51
*p*-value	0.001	$<$ 0.001	0.001	0.002	0.002

For clarity in the discussion, we denote RF as ${\mathrm{C}}_{1}$, XGBoost as ${\mathrm{C}}_{2}$, LightGBM as ${\mathrm{C}}_{3}$, SVM as ${\mathrm{C}}_{4}$, and the Stacked Ensemble as ${\mathrm{C}}_{5}$. As shown in the Table 9, the post-hoc pairwise comparisons revealed that the SVM (${\mathrm{C}}_{4}$) exhibited statistically significant differences when compared to all other classifiers (${\mathrm{C}}_{1}$–${\mathrm{C}}_{3}$ and ${\mathrm{C}}_{5}$) across all evaluation metrics, as indicated by consistently low *p*-values (*p* < 0.05). In contrast, many comparisons among the other classifiers did not show statistically significant differences. For instance, the performance of ${\mathrm{C}}_{1}$ vs ${\mathrm{C}}_{2}$ and ${\mathrm{C}}_{2}$ vs ${\mathrm{C}}_{3}$ was roughly on par, with *p*-values well above the 0.05 threshold, indicating no meaningful difference in those cases. Similar non-significant results are observed for other pairings that do not involve SVM, implying that the three tree-based models: ${\mathrm{C}}_{1}$, ${\mathrm{C}}_{2}$, and ${\mathrm{C}}_{3}$ performed comparably on this task. Overall, SVM achieved significantly higher accuracy, recall, specificity, and predictive values than its peers. This confirmed that the superior performance of the SVM was not due to random variation but rather reflects a genuine and substantial advantage over the competing models.

**Table 9:** *p*-values of Friedman post-hoc pairwise comparisons between classifiers across all evaluation metrics.

${\mathrm{C}}_{{\text{i }}} \;{\mathrm{vs}}\;{\mathrm{C}}_{{\mathrm{j}}}$	Friedman *p*-value				
	AC	RE	SP	PPV	NPV
${\mathrm{C}}_{1} \;{\mathrm{vs}}\;{\mathrm{C}}_{2}$	0.412	0.296	0.371	0.456	0.456
${\mathrm{C}}_{1} \;{\mathrm{vs}}\;{\mathrm{C}}_{3}$	0.332	0.233	0.296	0.296	0.296
${\mathrm{C}}_{1} \;{\mathrm{vs}}\;{\mathrm{C}}_{4}$	**0.007**	**0.007**	**0.007**	**0.007**	**0.007**
${\mathrm{C}}_{1} \;{\mathrm{vs}}\;{\mathrm{C}}_{5}$	0.551	0.765	0.456	0.551	0.551
${\mathrm{C}}_{2} \;{\mathrm{vs}}\;{\mathrm{C}}_{3}$	0.881	0.881	0.881	0.765	0.765
${\mathrm{C}}_{2} \;{\mathrm{vs}}\;{\mathrm{C}}_{4}$	< **0.001**	< **0.001**	< **0.001**	< **0.001**	< **0.001**
${\mathrm{C}}_{2} \;{\mathrm{vs}}\;{\mathrm{C}}_{5}$	0.156	0.179	0.101	0.179	0.179
${\mathrm{C}}_{3} \;{\mathrm{vs}}\;{\mathrm{C}}_{4}$	< **0.001**	< **0.001**	< **0.001**	< **0.001**	< **0.001**
${\mathrm{C}}_{3} \;{\mathrm{vs}}\;{\mathrm{C}}_{5}$	0.117	0.136	0.073	0.101	0.101
${\mathrm{C}}_{4} \;{\mathrm{vs}}\;{\mathrm{C}}_{5}$	**0.036**	**0.017**	**0.052**	**0.036**	**0.036**

Significant values are in [bold].

**Fig. 6:**
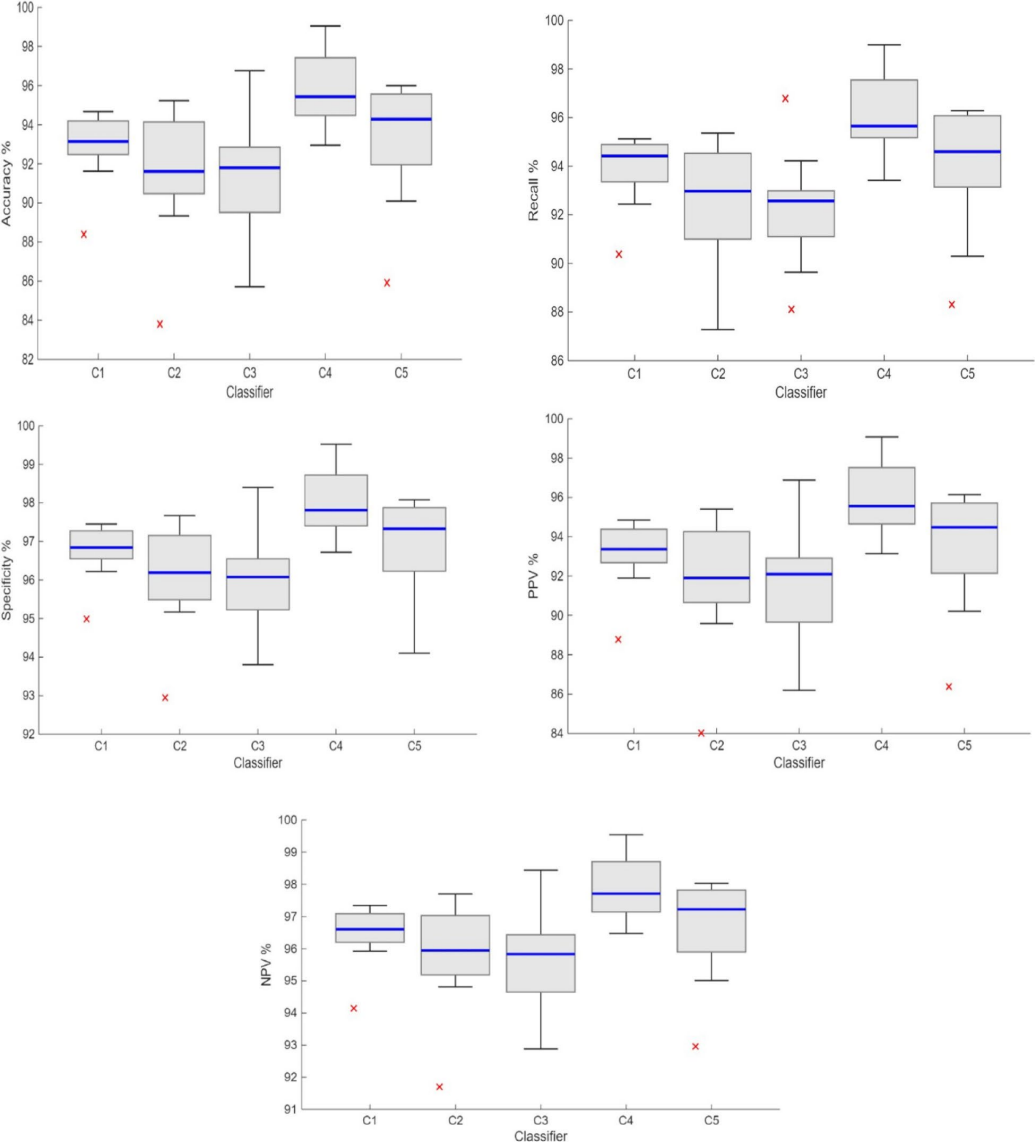
Boxplot comparison of classifiers performance across nine CGFF feature layers.

Figure 6 provides a visual summary of the performance distributions, showing boxplots for each metric across the five classifiers. Each boxplot represents the distribution of classifier performance across the nine CGFF feature layers, highlighting the median (blue central line), interquartile range (box edges), and range excluding outlier. Outliers are marked by red crosses to indicate cases that deviate notably from typical performance. Examining these boxplots across all evaluation metrics, SVM consistently demonstrated superior median performance compared to the other classifiers. This consistently high and stable performance across all metrics confirmed the Friedman test findings to select SVM as the optimal classifier for the brain tumor classification task in our study.

### Comparative analysis with SOTA approaches

Recent progress in brain tumor classification has produced promising results. However, several limitations persisted across existing approaches. Table 10 provides a comparative analysis with existing approaches from recent literature from SOTA models that were evaluated on the same dataset. The study of Gull et al.^38^ was limited by its reliance on traditional preprocessing (Gabor filtering and skull stripping) and generic feature extraction through a pre-trained VGG-19 model. These choices restrict the ability of the model to capture highly discriminative features and reduce generalization, which limited their classification accuracy to 98.25%. The approach of Farajzadeh et al.^39^ suffered from excessive preprocessing steps, dependence on handcrafted statistical features, reliance on synthetic data generation, and a highly resource-demanding architecture. Specifically, their pipeline integrated five consecutive image filtering operations followed by wavelet decomposition and statistical descriptors, which considerably increase computational burden and make the framework less practical for real-time use. Moreover, the dependence on manually engineered features reduced the generalization capacity of the model across heterogeneous datasets.

**Table 10:** A comparative analysis between the proposed model and SOTA on the same data source.

References	BraTs	Class	AC (%)	RE (%)	SP (%)	PPV (%)	NPV
2022^38^	2020	HGG/LGG	98.25	98	98.5	97.21	–
2023^39^	2020	HGG/LGG	98.93	98.93	–	98.93	–
2024^41^	2020	HGG/LGG	95.1	–	–	–	–
2025^42^	2020	HGG/LGG	80	84.09	63.64	90.24	–
2025^23^	2020	HGG/LGG/Nor	98.5	–	–	–	–
2025^44^	2020	HGG/LGG/Nor	98.66	99.04	–	99.25	–
2023^40^	2017	HGG/LGG/Nor	90.72	82.85	93.33	–	–
2024^10^	2017	HGG/LGG/Nor	98.88	98.66	99.00	–	–
Proposed model	2020	HGG/LGG/Nor	99.05	98.99	99.52	99.08	99.54

The model of Nazir et al.^41^ was computationally heavy, as it integrated an encoder with additional fully connected layers for multiple tasks. This design substantially increased training overhead, slows inference, and dilutes feature specialization for classification. Their method was restricted to an accuracy of 95.1%. The study by Liu et al.^42^ revealed significant limitations in adapting general-purpose text-based models to pixel-based medical tasks. Specifically, their models struggled with specificity and achieved notably lower accuracy compared to specialized deep learning architectures. The DCBT system introduced by Salama et al.^23^ utilized a transfer-learned EfficientNet-B0 backbone. The system relied on a single backbone architecture which limited the diversity of learned features compared to multi-model ensemble strategies that capture a broader range of semantic patterns. The classification pipeline of Topannavar et al.^44^ was sequentially dependent on a prior segmentation stage to isolate tumor regions. While this multi-stage dependency increased the computational complexity and makes the final classification performance contingent on the segmentation quality.

The framework of Ali et al.^40^ was constrained by its computationally heavy design, which integrated federated learning, CycleGAN-based domain mapping, and multi-stream CNNs. In addition, the reliance on 3D scan-level post-processing further increased training and inference costs, limiting its efficiency and clinical applicability. The reported accuracy of their method did not exceed 90.72%. The cascaded CNN architecture proposed by Abd-Ellah et al.^10^ employed multiple residual blocks, parallel local and global paths, and fully connected layers. This design made the system computationally heavy and less practical for real-time use. Moreover, extracting features across multiple paths might had introduced redundancy instead of improving discriminative ability. They also used fewer data samples compared with our study.

Overall, compared with the existing SOTA approaches, the proposed model demonstrated clear advantages that accounted for its superior performance. Unlike methods that relied on heavy preprocessing pipelines^39^, cascaded residual networks^10^, single-architecture backbones^23^, or federated multi-stream designs^40^, our framework adopted a streamlined yet powerful strategy. By contrast, the proposed framework introduced advanced preprocessing with AGC-DnCNN, which enhanced image quality and reduced noise effectively, and employed multi-CNN deep features fused with GLCM descriptors to capture both semantic and texture information. Furthermore, unlike VGG-based classifiers^38^, our model eliminated unnecessary preprocessing, avoided reliance on handcrafted descriptors or synthetic data generation, and adopted end-to-end deep feature learning with a more computationally efficient design. These methodological choices enabled the proposed system to surpass all compared models, achieving the highest accuracy of 99.05% together with high sensitivity, specificity, PPV, and NPV, thereby confirming its robustness, efficiency, and clinical applicability.

### Computational complexity and optimization

To assess the potential for translating the proposed framework into real-time clinical workflows, we evaluated the computational complexity of the best-performing network, TRENetB0 (layer 115). The inference time was calculated as the average processing duration per single MRI image. The processing pipeline entails varying computational demands across its stages. The preprocessing phase, utilizing the 59-layer DnCNN for image denoising and enhancement, represents the primary computational overhead, requiring an average of 0.70 s per image. In contrast, the feature extraction phase demonstrated the remarkable efficiency of the proposed TRENetB0. The forward pass through the TRENetB0 architecture combined with GLCM feature extraction is extremely fast, consuming only 0.022 s per image. This highlighted the lightweight nature of the proposed architecture. Finally, the classification stage via the SVM was computationally negligible, taking less than 0.001 s. Consequently, the total inference time for diagnosing a single MRI image was approximately 0.723 s. This sub-second processing time confirmed that the proposed framework was highly suitable for real-time computer-aided diagnosis without causing workflow delays.

Regarding optimization strategies, the proposed framework incorporated both algorithmic and operational refinements to maximize performance. First, at the training level, we employed adaptive optimization algorithms, specifically Adam, RMSprop, and SGDM to fine-tune the TRCNNs. Our results indicated that Adam generally provided the most stable convergence and superior accuracy for deep feature extraction, while RMSprop showed specific advantages in selected layers, justifying the use of a tailored optimization strategy for each network component. Second, to address the computational overhead in the preprocessing phase, specifically within the DnCNN, several optimization avenues are suggested for future clinical deployment. Since the 59-layer DnCNN consumes most of the inference time, employing model compression techniques is a practical solution. For instance, network pruning and model quantization can be utilized to lower the computational load. Quantization achieves this by reducing precision from 32-bit floating-point to 8-bit integers without compromising image quality. Furthermore, leveraging hardware acceleration would drastically decrease the processing time of the denoising stage. This can be implemented using high-performance GPUs with NVIDIA TensorRT or dedicated neural processing unit chips. These enhancements would ensure the system integrates seamlessly into daily medical workflows without processing delays.

## Conclusion

This study introduced a robust and efficient framework for brain tumor classification using MRI images, integrating deep learning and texture-based features to achieve superior diagnostic performance. The proposed algorithm began with an adaptive preprocessing stage utilizing ACG-DnCNN to enhance image clarity and suppress noise. Deep features were extracted from selected layers of three TRCNNs models, each optimized differently to capture diverse semantic representations. These were fused with rich texture descriptors to form nine CGFF sets that reflect both structural and contextual information. A comprehensive evaluation was conducted using five classifiers: RF, XGBoost, LightGBM, SVM, and a stacked ensemble. Among them, the SVM model trained on EfficientNetB0’s CGFF at layer 115 with the ADAM optimizer consistently delivered the best performance. On the BraTS 2020 dataset, it achieved 99.05% accuracy, 98.99% recall, 99.52% specificity, 99.08% PPV, and 99.54% NPV. The statistical significance of these findings was confirmed through Friedman tests and pairwise comparisons with *p*-values below 0.05. These results highlighted the effectiveness of combining deep semantic features with fine-grained textural details for accurate glioma classification. Future work will explore multi-class tumor subtype classification, domain generalization across institutions, and integration of the proposed model into real-time clinical workflows for enhanced decision support.

## Data Availability

The dataset in the manuscript is from public datasets: ([Multimodal Brain Tumor Segmentation Challenge 2020: Data | CBICA | Perelman School of Medicine at the University of Pennsylvania] (https://www.med.upenn.edu/cbica/brats2020/data.html)).

## References

[1] Khalil, H. F. et al. CNN-MR tumor classifier: Brain tumors classification system based on CNN transfer learning models combined with distributed computing process. *J. Adv. Eng. Trends* **43**, 399–423. 10.21608/jaet.2024.237567.1259 (2024).

[2] Kuang, Z. et al. Global disease burden, trends, and inequalities of brain and central nervous system cancers, 1990–2021: A population-based study with projections to 2036. *World Neurosurg.* **198**, 123970 (2025). 40274017 10.1016/j.wneu.2025.123970

[3] Nassar, S. E., Yasser, I., Amer, H. M. & Mohamed, M. A. A robust MRI-based brain tumor classification via a hybrid deep learning technique. *J. Supercomput.* **80**, 2403–2427 (2024).

[4] Shahi, M. & Cooks, R. G. Ambient ionization mass spectrometry in brain cancer diagnosis. *J. Mass Spectrom. Adv. Clin. Lab* (2025). 10.1016/j.jmsacl.2025.10.002 PMC12590441 41211508

[5] van Zandvoort, M. J., Snijders, T. J., Oomens, J. E., van Kessel, E. & Robe, P. A. J. T. *Drivers and Consequences of Cognitive Functioning in Glioma*, Vol. 203 (2022).

[6] Koçak, M., Atasoy, Ö., Çini, N. & Erbaş, O. Current trends in Glioblastoma. *Demiroglu Sci. Univ. Florence Nightingale J. Med.* **7**, 314–322 (2021).

[7] Agarwal, M. et al. Deep learning for enhanced brain Tumor Detection and classification. *Results Eng.* **22**, 102117 (2024).

[8] Bayoumi, E., Khalaf, A. A. & Gharieb, R. R. Brain tumor automatic detection from MRI images using transfer learning model with deep convolutional neural network. *J. Adv. Eng. Trends* **41**, 19–30 (2021).

[9] Ibrahim, A. M., Rahouma, K. & Hamed, H. F. A. Deep neural network for breast tumor classification through histopathological image. *J. Adv. Eng. Trends* **42**, 121–129. 10.21608/jaet.2021.67697.1099 (2022).

[10] Abd-Ellah, M. K., Awad, A. I., Khalaf, A. A. & Ibraheem, A. M. Automatic brain-tumor diagnosis using cascaded deep convolutional neural networks with symmetric U-Net and asymmetric residual-blocks. *Sci. Rep.* **14**, 9501 (2024). 38664436 10.1038/s41598-024-59566-7 PMC11045751

[11] McBee, M. P. et al. Deep learning in radiology. *Acad. Radiol.* **25**, 1472–1480 (2018). 29606338 10.1016/j.acra.2018.02.018

[12] Mansour, R. F. et al. Artificial intelligence with big data analytics-based brain intracranial hemorrhage e-diagnosis using CT images. *Neural Comput. Appl.* **35**, 16037–16049 (2023).

[13] Özcan, H. et al. A comparative study for glioma classification using deep convolutional neural networks. *Mol. Biol. Evol.* (2021). 10.3934/mbe.2021080 33757198

[14] Arabahmadi, M., Farahbakhsh, R. & Rezazadeh, J. Deep learning for smart Healthcare—A survey on brain tumor detection from medical imaging. *Sensors* **22**, 1960 (2022). 35271115 10.3390/s22051960 PMC8915095

[15] Ahmed, H. A., Rahouma, K. H. & Massoud, M. A. Automated detection of primary liver cancer using different deep learning approaches. *J. Adv. Eng. Trends* **43**, 433–449. 10.21608/jaet.2024.255537.1269 (2024).

[16] Lundervold, A. S. & Lundervold, A. An overview of deep learning in medical imaging focusing on MRI. *Z. Med. Phys.* **29**, 102–127 (2019). 30553609 10.1016/j.zemedi.2018.11.002

[17] Arora, G., Dubey, A. K., Jaffery, Z. A. & Rocha, A. A comparative study of fourteen deep learning networks for multi skin lesion classification (MSLC) on unbalanced data. *Neural Comput. Appl.* **35**, 7989–8015 (2023).

[18] Morovati, B., Lashgari, R., Hajihasani, M. & Shabani, H. Reduced deep convolutional activation features (r-decaf) in histopathology images to improve the classification performance for breast cancer diagnosis. *J. Digit. Imaging* **36**, 2602–2612 (2023). 37532925 10.1007/s10278-023-00887-w PMC10584742

[19] Tesfai, H. et al. Lightweight shufflenet based CNN for arrhythmia classification. *IEEE Access* **10**, 111842–111854 (2022).

[20] Bekheet, M. et al. Cardiac fibrosis automated diagnosis based on FibrosisNet network using CMR ischemic cardiomyopathy. *Diagnostics* **14**, 255 (2024). 38337771 10.3390/diagnostics14030255 PMC10855193

[21] Fortuna-Cervantes, J. M. et al. Texture and materials image classification based on wavelet pooling layer in CNN. *Appl. Sci.* **12**, 3592 (2022).

[22] Dheepak, G. & Vaishali, D. Brain tumor classification: A novel approach integrating GLCM, LBP and composite features. *Front. Oncol.* **13**, 1248452 (2024). 38352298 10.3389/fonc.2023.1248452 PMC10861642

[23] Salama, G. M. et al. Brain tumor diagnosis techniques key achievements, lessons learned, and a new CNN architecture. *Egypt. J. Radiol. Nucl. Med.* **56**, 166. 10.1186/s43055-025-01567-1 (2025).

[24] Toğaçar, M., Ergen, B. & Cömert, Z. BrainMRNet: Brain tumor detection using magnetic resonance images with a novel convolutional neural network model. *Med. Hypotheses* **134**, 109531. 10.1016/j.mehy.2019.109531 (2020). 31877442 10.1016/j.mehy.2019.109531

[25] Tandel, G. S., Tiwari, A. & Kakde, O. G. Performance optimisation of deep learning models using majority voting algorithm for brain tumour classification. *Comput. Biol. Med.* **135**, 104564 (2021). 34217980 10.1016/j.compbiomed.2021.104564

[26] Alanazi, M. F. et al. Brain tumor/mass classification framework using magnetic-resonance-imaging-based isolated and developed transfer deep-learning model. *Sensors* **22**, 372 (2022). 35009911 10.3390/s22010372 PMC8749789

[27] Younis, A., Qiang, L., Nyatega, C. O., Adamu, M. J. & Kawuwa, H. B. Brain tumor analysis using deep learning and VGG-16 ensembling learning approaches. *Appl. Sci.* **12**, 7282 (2022).

[28] Alsaif, H. et al. A novel data augmentation-based brain tumor detection using convolutional neural network. *Appl. Sci.* **12**, 3773 (2022).

[29] Salama, W. M. & Shokry, A. A novel framework for brain tumor detection based on convolutional variational generative models. *Multimed. Tools Appl.* **81**, 16441–16454 (2022).

[30] Srinivas, C. et al. Deep transfer learning approaches in performance analysis of brain tumor classification using MRI images. *J. Healthc. Eng.* **2022**, 3264367 (2022). 35299683 10.1155/2022/3264367 PMC8923754

[31] Asiri, A. A. et al. Machine learning-based models for magnetic resonance imaging (MRI)-based brain tumor classification. *Intell. Autom. Soft Comput* **36**, 299–312 (2023).

[32] Salama, G. M., Ashraf, S., Bayoumi, E. S., Elwan, M. & Abd-Ellah, M. K. in *2024 International Telecommunications Conference (ITC-Egypt).* 72–77 (IEEE).

[33] Chukwujindu, E., Faiz, H., Sara, A.-D., Faiz, K. & De Sequeira, A. Role of artificial intelligence in brain tumour imaging. *Eur. J. Radiol.* **176**, 111509 (2024). 38788610 10.1016/j.ejrad.2024.111509

[34] Hemanvitha, K. & Dhiman, V. Leveraging predictive analytics: Enhancing brain tumor classification with xgboost. In *The Impact of Algorithmic Technologies on Healthcare*, 85–101 (2025).

[35] Mzoughi, H. et al. Deep multi-scale 3D convolutional neural network (CNN) for MRI gliomas brain tumor classification. *J. Digit. Imaging* **33**, 903–915 (2020). 32440926 10.1007/s10278-020-00347-9 PMC7522155

[36] Ge, C., Gu, I.Y.-H., Jakola, A. S. & Yang, J. Deep semi-supervised learning for brain tumor classification. *BMC Med. Imaging* **20**, 1–11 (2020). 10.1186/s12880-020-00485-0 PMC7391541 32727476

[37] El Hamdaoui, H. et al. High precision brain tumor classification model based on deep transfer learning and stacking concepts. *Indones. J. Electr. Eng. Comput. Sci* **24**, 167–177 (2021).

[38] Gull, S., Akbar, S., Hassan, S. A., Rehman, A. & Sadad, T. in *International Conference on Emerging Technology Trends in Internet of Things and Computing.* 182–194 (Springer).

[39] Farajzadeh, N., Sadeghzadeh, N. & Hashemzadeh, M. Brain tumor segmentation and classification on MRI via deep hybrid representation learning. *Expert Syst. Appl.* **224**, 119963 (2023).

[40] Ali, M. B., Gu, I.Y.-H., Berger, M. S. & Jakola, A. S. A novel federated deep learning scheme for glioma and its subtype classification. *Front. Neurosci.* **17**, 1181703 (2023). 37287799 10.3389/fnins.2023.1181703 PMC10242007

[41] Nazir, M., Shakil, S. & Khurshid, K. End-to-end multi-task learning architecture for brain tumor analysis with uncertainty estimation in MRI images. *J. Imaging Inform. Med.* **37**, 2149–2172 (2024). 38565728 10.1007/s10278-024-01009-w PMC11522262

[42] Liu, F., Yoo, J. J. & Khalvati, F. A Comparison and evaluation of fine-tuned convolutional neural networks to large language models for image classification and segmentation of brain tumors on MRI. arXiv preprint http://arxiv.org/abs/2509.10683 (2025).

[43] Berghout, T. The neural frontier of future medical imaging: A review of deep learning for brain tumor detection. *J. Imaging* **11**, 2 (2024). 39852315 10.3390/jimaging11010002 PMC11766058

[44] Topannavar, P. S., Bendre, V. S. & Khurge, D. Accurate brain tumor classification with STN-NAM in ResNet50 using MRI. *Bull. Electr. Eng. Inform.* **14**, 1241–1250 (2025).

[45] Huang, G., Liu, Z., Van Der Maaten, L. & Weinberger, K. Q. in *Proceedings of the IEEE Conference on Computer Vision and Pattern Recognition.* 4700–4708.

[46] Raiaan, M. A. K. et al. A systematic review of hyperparameter optimization techniques in Convolutional Neural Networks. *Decis. Anal. J.* **11**, 100470 (2024).

[47] Gurunathan, A. & Krishnan, B. A hybrid CNN-GLCM classifier for detection and grade classification of brain tumor. *Brain Imaging Behav.* **16**, 1410–1427 (2022). 35048264 10.1007/s11682-021-00598-2

[48] Mohammadagha, M. Hyperparameter optimization strategies for tree-based machine learning models prediction: A comparative study of AdaBoost, decision trees, and random forest. In *Decision Trees, and Random Forest (April 11, 2025)* (2025).

[49] Jogunuri, S. et al. Random forest machine learning algorithm based seasonal multi-step ahead short-term solar photovoltaic power output forecasting. *IET Renew. Power Gener.* **19**, e12921 (2025).

[50] Chen, T. & Guestrin, C. in *Proceedings of the 22nd ACM SIGKDD International Conference on Knowledge Discovery and Data Mining*, 785–794.

[51] Jabardi, M. Support vector machines: Theory, algorithms, and applications. *Infocommun. J.* **17** (2025).

[52] Md, A. Q. et al. Enhanced preprocessing approach using ensemble machine learning algorithms for detecting liver disease. *Biomedicines* **11**, 581 (2023). 36831118 10.3390/biomedicines11020581 PMC9953600

[53] Jaiyeoba, O., Ogbuju, E., Yomi, O. T. & Oladipo, F. Development of a model to classify skin diseases using stacking ensemble machine learning techniques. *J. Comput. Theor. Appl.* **2**, 22–38 (2024).

[54] Menze, B. H. et al. The multimodal brain tumor image segmentation benchmark (BRATS). *IEEE Trans. Med. Imaging* **34**, 1993–2024 (2014). 25494501 10.1109/TMI.2014.2377694 PMC4833122

[55] Setiadi, D. R. I. M. PSNR vs SSIM: Imperceptibility quality assessment for image steganography. *Multimed. Tools Appl.* **80**, 8423–8444 (2021).

[56] Rahman, A. B., Afjal, M. I. & Mamun, M. A. A. Systematic evaluation of wavelet-based denoising for MRI brain images: Optimal configurations and performance benchmarks. arXiv preprint http://arxiv.org/abs/2508.15011 (2025).

[57] Mowla, M. R., Gonzalez-Morales, J. D., Rico-Martinez, J., Ulichnie, D. A. & Thompson, D. E. A comparison of classification techniques to predict brain–computer interfaces accuracy using classifier-based latency estimation. *Brain Sci.*10.3390/brainsci10100734 (2020). 33066374 10.3390/brainsci10100734 PMC7602195

